# Dynamic Collision Fingerprints (DCF): Introducing a New Descriptor Linking Lattice Interactions to 2D Structural Data Signatures

**DOI:** 10.1021/acs.jctc.5c00856

**Published:** 2025-08-12

**Authors:** Raphael M. Tromer

**Affiliations:** Institute of Physics, 28127 University of Brasília, Brasília, Federal District 70910-900, Brazil

## Abstract

We introduce a novel method for structural characterization of two-dimensional (2D) materials based on classical particle trajectories and elastic collisions with atomic lattices. This approach, called the dynamic collision fingerprint (DCF), encodes structural features through statistical patterns of collisions, capturing symmetry, porosity, and disorder using purely geometric information. Applied to systems such as graphene, phagraphene, CEY-graphene, and h-BN, the method extracts descriptors, including mean free path, diffusivity, angular entropy, and Fourier-based symmetry metrics. These form compact interpretable vectors suitable for classification and machine learning. All calculations were performed on a personal computer, underscoring the method’s efficiency and accessibility. DCF provides a robust and general tool for 2D materials analysis, particularly well-suited for carbon-based and atomically thin systems.

## Graphical abstract

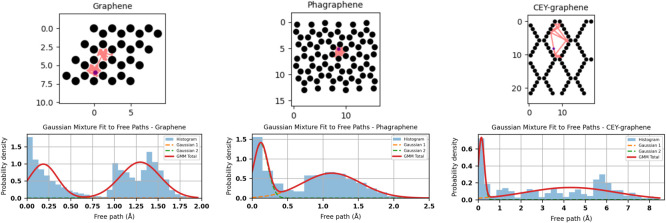

## Introduction

The understanding and structural characterization of materials,^1–3^ especially those with reduced dimensionality such as two-dimensional (2D) systems,^4–7^ is a central element in predicting physicochemical properties, enabling the rational design of new compounds,^8,9^ and supporting the development of emerging technological applications.^10,11^ Since the discovery of graphene and the subsequent surge of interest in 2D materials,^12,13^ the need for tools capable of objectively and automatically describing,^14–16^ quantifying,^17,18^ and comparing their structures has become increasingly apparent.

In this context, structural descriptors,^19–21^ variables or sets of variables that encode relevant information about the geometry,^22^ symmetry,^23^ order or disorder^24,25^ of a system, play a fundamental role. They serve as bridges between the atomic-scale structure and emergent properties, being essential in fields such as atomistic modeling,^26^ machine learning for materials science,^27–29^ structural phase classification,^30^ and transition analysis.^31^

These descriptors can take multiple forms. Some are based on explicit atomic coordinates,^32^ such as radial distribution functions (RDFs),^33^ angular distributions,^34^ or orientational correlation functions.^35^ Others rely on global properties of the system, such as point and translational symmetries,^36^ measures of configurational entropy,^37^ or spectral analysis of bonding graphs.^38^ More modern approaches include machine learning-oriented descriptors such as SOAP (smooth overlap of atomic positions),^39^ ACSF (atom-centered symmetry functions),^40^ and graph neural network-based descriptors,^41^ which learn representations directly from structural data.^42^

Despite this diversity and sophistication, many of these descriptors share common limitations: they require highly accurate (often idealized) atomic positions, are sensitive to thermal perturbations or disorder,^35^ and frequently depend on complex parametrizations or specific basis functions.^43^ Additionally, most assume a fundamentally static view of structure, capturing a “snapshot” of atomic organization, which may obscure dynamic or emergent aspects only revealed under interaction with external agents.^39,42^

In this work, we propose a conceptually different approach: rather than describing structure through direct coordinates or explicit symmetries, we aim to capture how a structure dynamically interacts with simple external particles, through a model inspired by classical statistical mechanics.^44^ The model can be visualized as an adapted version of the Drude–Galton system: rigid spheres (balls) are launched toward a periodic 2D structure, representing the material, and undergo elastic collisions with its components (atoms or scattering sites).^45–47^ The focus here, however, is not on particle conduction or diffusion but on the geometric statistics of the resulting collisions.

Our guiding hypothesis is that collision patterns, such as the distribution of distances between successive impacts, the orientation of collision normals, or angular recurrence in complex trajectories, subtly yet consistently reflect the internal organization of the structure. In other words, the structure “responds” to the passage of particles, and this response can be quantified objectively, yielding a dynamic descriptor sensitive to order, symmetry, and complexity without relying on electronic models, energetic considerations, or external parameters.

This is thus a proposal that combines implementation simplicity, geometric intuition, and generalization potential: any 2D structure, from perfectly periodic crystals to disordered or quasi-crystalline arrangements, can be subjected to this model, and the resulting statistics can serve as unique signatures or fingerprints of the structure. This offers a new interpretative layer for structural characterization that is potentially complementary to traditional descriptors.

Furthermore, by exploring purely mechanical collisions, we open the door to physical interpretations that, while stylized, remain grounded in real interactions. These can be visualized, statistically analyzed, and eventually correlated with the relevant material properties. We refer to this set of collected information as the dynamic collision fingerprint (DCF), a structural fingerprint derived solely from the statistical response of mechanical collisions. Variables involved may include, for instance, the distribution of distances between successive collisions, the angular orientation of impact normals, the angular density of events, and directional recurrence across multiple trajectories. The guiding principle is that such patterns are not random: they are shaped by the structure’s internal organization and thus encode meaningful information about its symmetry, order, complexity, and even disorder.

The DCF descriptors are distinguished by several key characteristics: (i) Independence from electronic, thermal, or chemical models, as the structure is analyzed purely through its geometry. (ii) Physical and computational simplicity, where the model can be implemented efficiently and visualized intuitively. (iii) Emergent sensitivity, in which similar structures produce similar signatures, while distinct geometries yield distinguishable fingerprints. (iv) Generality and robustness, as the methodology applies to crystalline, amorphous, or quasi-periodic systems alike.

We believe that DCF descriptors represent both conceptual and practical contributions to materials science. They offer a novel, dynamic, geometric, and interpretable route to understand and compare two-dimensional structures. In this paper, we detail the theoretical foundations of the DCF methodology, discuss its construction principles, and demonstrate, through illustrative examples, how different structures produce distinct fingerprints. We thus propose a complementary tool to traditional methods, with potential applications in structural classification, phase transition analysis, and ultimately in automated pipelines for materials discovery.

In addition to their role in structural characterization, descriptors are fundamental for enabling machine learning models that predict physicochemical properties and guide materials discovery, particularly for 2D systems. Recent studies have employed structural descriptors such as radial and angular distributions, orientational order parameters, and machine learning-oriented representations such as SOAP or graph-based embeddings to establish structure–property relationships. However, these approaches often face challenges when dealing with complex, disordered, or thermally perturbed structures, which are common in two-dimensional materials. Motivated by these limitations, DCF offers a complementary strategy: a geometry-based, physically grounded descriptor that captures emergent structural information beyond static atomic positions. As such, it holds potential not only for structural classification but also as an interpretable, robust input for property prediction frameworks. As will be discussed in the following sections, one of the main contributions of DCF is its ability to capture the angular order of two-dimensional systems in a simple and efficient manner. In future applications, this approach may be extended to investigate how factors such as vacancy or substitutional disorder affect the angular symmetry and structural order of 2D crystals. Furthermore, the flexibility of the DCF model allows for the straightforward inclusion of chemical identity or other relevant system-specific features, broadening its applicability without sacrificing simplicity or physical intuition.

## Methodology

This work proposes and applies a simple and geometrically intuitive computational method for the structural characterization of two-dimensional (2D) atomic arrangements. The approach is based on a classical simulation of the trajectory of a particle (“ball”) moving through a fixed array of circular obstacles (“pins”), whose positions are directly derived from atomic coordinates extracted from crystallographic information files (CIF format). The method combines implementation simplicity, geometric intuition, and remarkable generalization potential: any 2D structure, from perfectly periodic crystals to disordered or quasi-crystalline arrangements, can be subjected to this model. The statistics resulting from the ball dynamics function as unique structural signatures (or fingerprints), potentially complementary to conventional descriptors used in materials characterization.

To establish a model based on a Galton board framework, we first define the geometry of the medium and the definition of pins. The simulation is based on a set of atomic positions extracted from a CIF file loaded using the Atomic Simulation Environment (ASE) library. The unit cell is replicated along *X*, *Y*, and *Z* according to *N*_*X*_ × *N*_*Y*_ × 1, generating a 2D periodic arrangement in the *XY* plane. Each atom in the replicated structure is treated as the center of a circular pin. The radius of each pin, *r*_pin_, is determined from the atomic radius relative to carbon, according to

1 $$r_{\mathrm{pin}}=\frac{r_{\mathrm{atomic}}}{r_{C}}\times r_{\mathrm{pin}}^{\mathrm{ref}}$$

where *r*_atomic_ is the atomic radius of the corresponding element, *r*_C_ is the atomic radius of carbon (used as a reference), and *r*_pin_^ref^ is an arbitrary scaling factor. This normalization ensures consistency across different atom types and provides a relative scale coherent with the local geometry of the system. The simulation tracks the trajectory of a single spherical particle (a ball of mass *m*_b_) with a fixed radius, although multiple simultaneous launches can be performed to explore a larger portion of the available free path. The ball does not have a physically realistic mass; instead, we adopt an artificial mass for numerical convenience, expressed in units of eV·ps^2^/Å^2^. This choice ensures compatibility with the unit system (distances in Å, time in picoseconds, and energy in eV) and allows for the definition of a thermal velocity without compromising the robustness of the model. The ball is launched from a random position on the board with an initial velocity calculated from the classical average kinetic energy at *T* = 300 K.

This sets up a collision dynamics, where the ball’s trajectory is updated at fixed time steps Δ*t* = 0.1 ps, over *N*_steps_. The motion is uniform and rectilinear (no acceleration), except during collisions. Collisions with pins are treated as elastic reflections on circular surfaces: the ball’s velocity is reflected around the local normal at the impact point, and its position is adjusted to remain outside the overlapping region. The simulation uses periodic boundary conditions in the (*x*, *y*) plane, meaning the ball that exits one boundary of the cell re-enters from the opposite side (torus topology).

For each structural configuration, we perform N independent launches, each accepted only if at least two collisions occur. From the validated trajectories, we extract: free paths λ from the distances between successive collisions, relaxation times τ (time intervals between collisions), effective diffusion coefficient, given by *D* = ⟨λ⟩^2^/2⟨τ⟩,^48^ and collision angles defined relative to the local normal at the point of contact.

Figure 1 shows representative snapshots of the atomic configurations used in this work, graphene, phagraphene, and CEY, along with sample trajectories of the tracer particle (red) interacting with the scattering centers (gray) (see movies in the Supporting Information). The radii of the tracer and the obstacles were set to 0.50(0.25) Å for pin­(ball), values which are used consistently throughout the study, as further discussed in the parameter selection analysis in the next section. Under these conditions, the tracer tends to experience local confinement within individual rings or pores, leading to trajectories that remain within a given structural motif for several collisions. This behavior is a key strength of the DCF approach as it allows the descriptor to capture the geometric and angular characteristics of each ring type. By initializing tracer trajectories from random positions across the entire simulation domain, we ensure that all local environments, each with its own symmetry and topological signature, contribute to the final fingerprint. This strategy results in a comprehensive and interpretable structural representation, particularly well-suited for systems that exhibit a diversity of pore geometries such as phagraphene and CEY-graphene.

**Figure 1:**
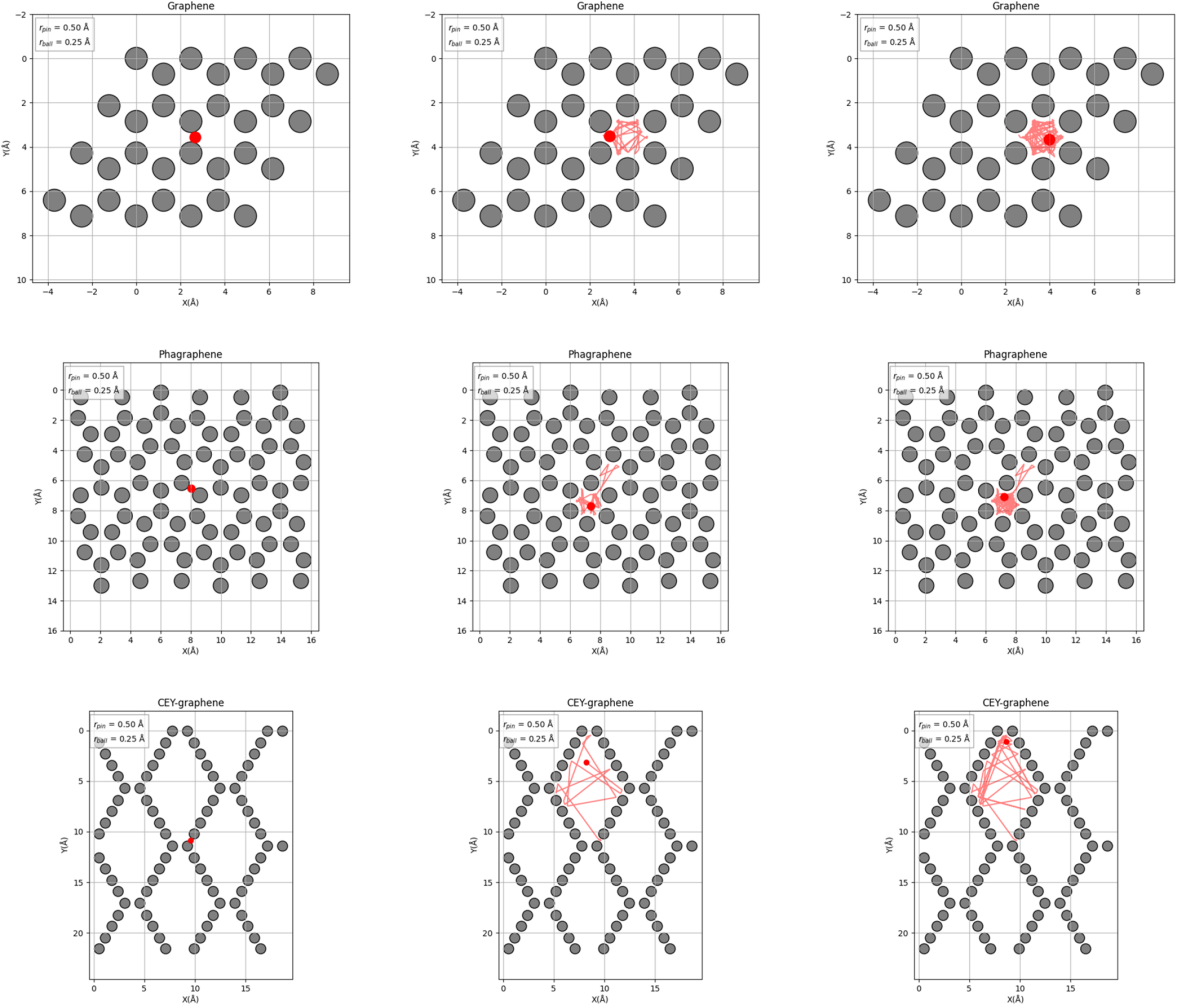
Snapshots of the atomic structures and representative tracer trajectories used in the DCF simulations for three 2D materials: graphene (top row), phagraphene (middle row), and CEY (bottom row). Gray circles represent the atomic scattering centers (pins), while the red dot marks the initial position of the tracer particle, and red lines indicate segments of its elastic trajectory. All simulations were performed with *r*_pin_ = 0.50 Å and *r*_ball_ = 0.25 Å, as annotated. The chosen size ratio induces local confinement of the tracer within rings or pores, enabling the DCF to capture the angular and topological features of distinct structural motifs. Randomized initial conditions across the structure ensure that the final fingerprint incorporates all of the relevant environments.

The distribution of collision angles is used to infer symmetry properties of the underlying structure, modeled through Shannon entropy, $S=-\sum_{i}p_{i}\log\left(p_{i}\right)$ ,^49^ normalized by log­(*N*_bins_), where *p*_*i*_ is the relative frequency in each angular bin and *S*_norm_ ∈ [0, 1].

To complement this measure, we apply the fast Fourier transform (FFT)^50^ to the angular histogram. The magnitude spectrum of the FFT reveals periodic features in the angular distribution, identifying dominant rotational symmetries (e.g., 6-fold, 10-fold). The intensity of each harmonic component is normalized by the zero-frequency (DC) term, providing a rotational order spectrum that quantifies the strength of the angular ordering. These two metrics capture distinct but complementary structural characteristics: (i) Entropy measures the overall randomness in angular distribution, ideal for distinguishing highly disordered (amorphous) configurations. (ii) FFT intensity detects periodic angular features, which entropy alone cannot resolve, making it suitable for identifying ordered or quasi-crystalline patterns. Based on these combined criteria, we classify the structures as follows:

- Amorphous: high angular entropy (*S*_norm_ > 0.8);
- Ordered: strong peak in dominant harmonic (*S*_norm_ < 0.8 and order intensity >0.1);
- Quasi-crystalline: moderate peak *S*_norm_ < 0.8 and order intensity about (0.05 – 0.1);
- Disordered: no clear patterns.

This classification of the structures was defined by us, assigning a similar weight to each crystalline phase. Since our primary goal is to compare different structures, this definition is arbitrary—as long as we consistently apply the same rule to the structural descriptors proposed in this work.

These considerations form a type of geometric fingerprint of the structure, offering a novel interpretive layer for structural analysis complementary to traditional descriptors (e.g., RDF, structure factor, point symmetry).

## Results

The initial stage of our analysis focused on the selection and justification of the key parameters governing the simulation, aiming to ensure a balance among physical consistency, computational tractability, and statistical representativeness of the resulting fingerprints. We began by investigating the appropriate value for the mass of the probe particle (“ball”). Since this mass does not correspond to any real atom, it was defined in arbitrary units suitable for compatibility with the simulation’s unit system (distances in Å, time in ps, and energy in eV). A value of *m*_b_ = 1.0 eV ps^2^/Å^2^ was adopted, which, at room temperature (*T* = 300 K), yields a thermal velocity of approximately 0.227 Å/ps. This choice was empirically validated as it provides a time scale compatible with the typical interpin distances, ensuring that trajectories remain sensitive to structural details while avoiding excessive particle trapping or erratic behavior.

To define the scattering centers (pins), we introduced a normalized radius based on the atomic radius of each element relative to that of carbon (see eq 1). This approach ensures that each atom contributes proportionally to its physical size, allowing for heterogeneous atomic environments while preserving geometric consistency. The normalization by the carbon radius serves as a practical reference, especially given its prominence in many 2D materials.

We initially tested the model on a minimal system created by a 4 × 4 × 1 replication of the graphene unit cell, which allowed rapid testing of parameters, verification of periodic boundary conditions, and visual inspection of the trajectories.

To ensure statistical reliability, we performed 100 independent launches for each configuration, each consisting of 10,000 time steps with a fixed interval of Δ*t* = 0.1 ps, corresponding to a total trajectory duration of 1000 ps per particle. This configuration proved to be effective in producing a representative sample of collision events. Most trajectories resulted in at least two valid collisions, allowing for the computation of meaningful statistics on free path lengths (λ), relaxation times (τ), and effective diffusion coefficients *D*. The distributions generated from these trajectories form the basis of the structural fingerprints proposed in this study.

First, we aim to determine and establish an appropriate value for parameter *N*_step_, which represents the number of steps of each particle in the simulations. To this end, we performed simulations with different values of *N*_step_, ranging from 1 × 10^2^ to 1 × 10^6^, in order to identify the point at which the relevant physical quantities converge. A replicated graphene cell with dimensions 4 × 4 × 1 was used along the *x*, *y*, and *z* directions, respectively. The geometric parameters of the system were kept fixed, with the radius of the pin set to 0.5 Å and the ball set to 0.25 Å. Figure 2 shows the variation of the mean free path λ, the relaxation time τ, and the diffusion coefficient *D* as functions of the number of steps *N*_step_. It is observed that for small values of nstep (such as 10^2^ and 10^3^), the three quantities exhibit significant oscillations, indicating that the system has not yet reached a stationary regime. These deviations are due to initial correlation effects and limited sampling statistics. Starting from approximately 10^4^ steps, the values of λ, τ, and *D* stabilize and show only minor fluctuations. This behavior indicates that the system has reached the statistical equilibrium necessary for the reliable sampling of the transport properties. Based on these results, we adopted *N*_step_ = 10^4^ as the standard value for the subsequent simulations, as it provides a balance between computational efficiency and statistical accuracy.

**Figure 2:**
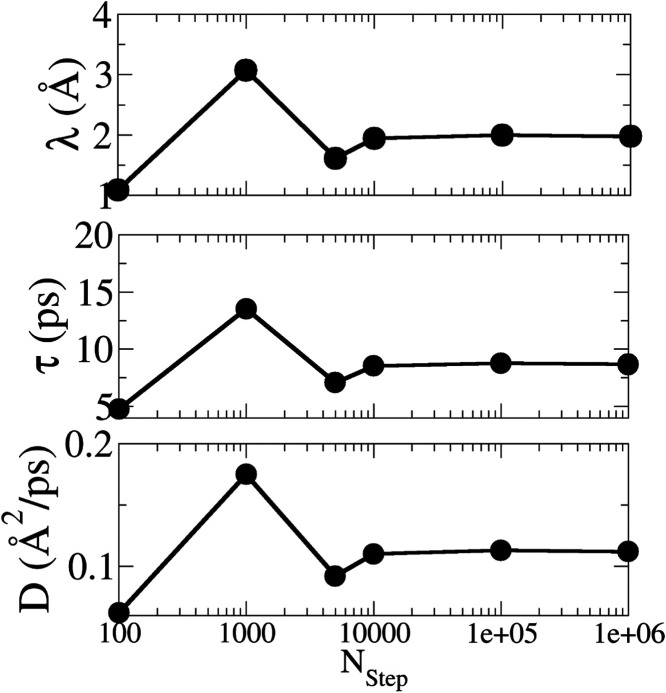
Dependence of the mean free path λ (top), relaxation time τ (middle), and diffusion coefficient *D* (bottom) as a function of the number of steps *N*_step_. The stabilization of the parameters beyond 10^4^ steps indicates that this value ensures convergence and reliable statistical sampling of the transport properties.

Since the quantities investigated here are subject to the same influence of external parameters, as shown in Figure 2, we will use only the diffusion coefficient as a metric to determine the most appropriate values for the radii *r*_ball_ and *r*_pin_. In Figure 3a, we present *D* as a function of *r*_ball_, keeping *r*_pin_ fixed. It can be observed that for low values of *r*_pin_, the diffusion coefficient decreases smoothly with increasing *r*_ball_ until it reaches a plateau, indicating a regime in which the angular phase becomes well-defined. However, although this condition favors the angular description of the system, very high values of *r*_pin_ may compromise the visualization of the structure through collisions, due to excessive overlap and noise in the impact flow, as shown in Figure 3c.

**Figure 3:**
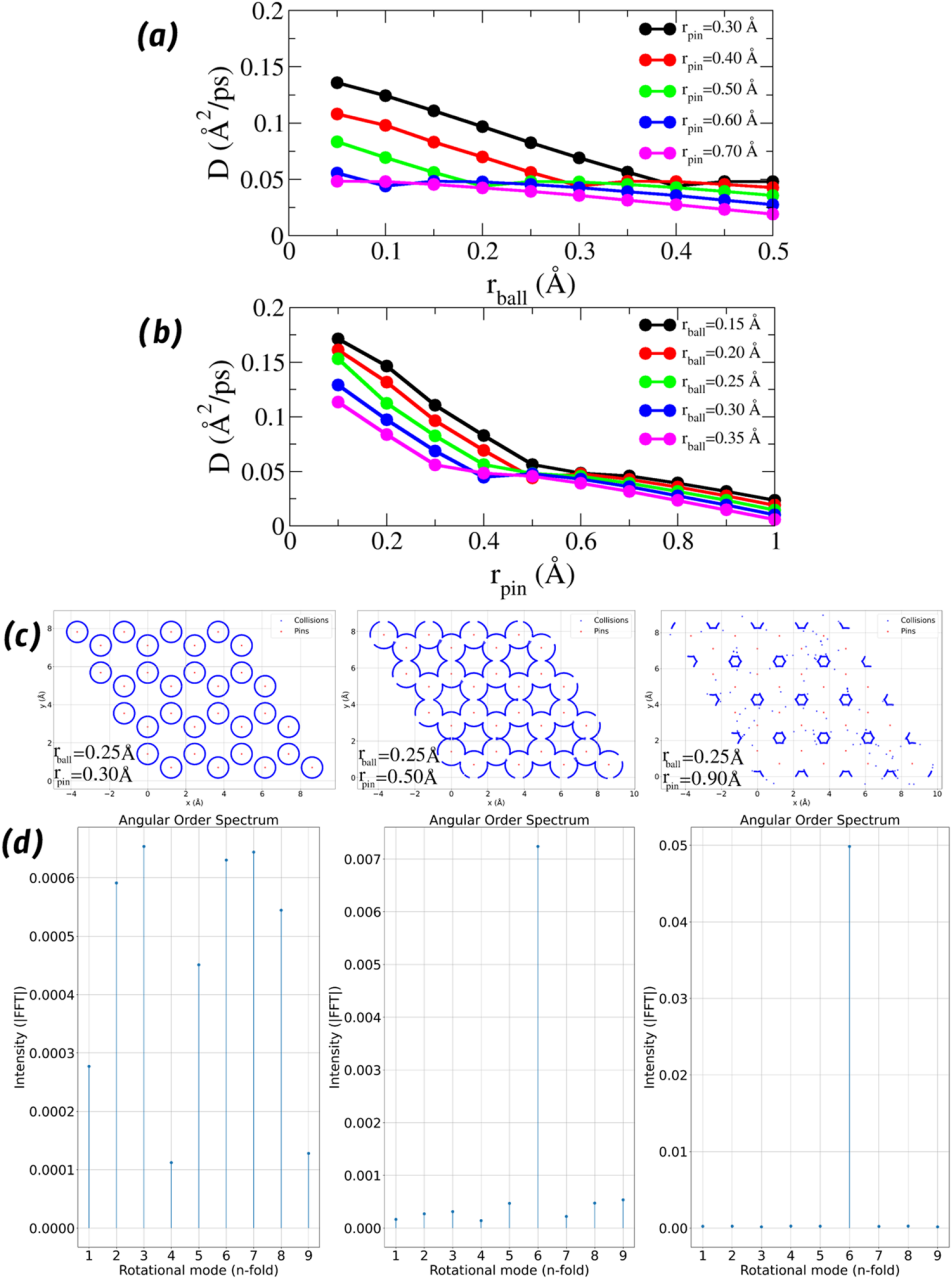
Influence of geometric parameters on diffusion, angular order, and structural visualization. (a) Diffusion coefficient *D* as a function of ball radius *r*_ball_, for different fixed pin radii *r*_pin_. (b) *D* as a function of *r*_pin_, with a fixed *r*_ball_. The curve inflection points reveal a regime transition, guiding optimal parameter selection. (c) Collision maps for three representative parameter combinations, illustrating the trade-off between structural clarity and angular order. (d) Corresponding angular order spectra, where a dominant rotational mode emerges when *D* stabilizes.

In Figure 3b, we observe a clearer analysis of the regime transition: *D* is shown as a function of *r*_pin_, with *r*_ball_ fixed at different values. The curves reveal an inflection region or “elbow,” characterized by a change in the behavior of *D*, from a rapid variation to an almost constant regime, followed again by a decrease. This transition indicates the emergence of a collision geometry that simultaneously captures the angular order of the system and allows a coherent spatial visualization of the structure.

Thus, we propose that the optimal parameters lie near this inflection point (the transition region between regimes), as they represent an optimal trade-off between accuracy in the angular description and fidelity in the structural reconstruction based on collisions. These values avoid both angular degeneracy (associated with highly variable *D*) and geometric noise (associated with overly wide pins). This approach enables the extraction of structural properties with greater physical realism and visual clarity.

In this work, we will use values of 0.25 Å for *r*_ball_ and 0.50 Å for *r*_pin_ in our simulations, which were found to offer a good compromise in satisfactorily describing both the orientational order and the structure of each analyzed system, as shown in Figure 3c,d, where red dots indicate the positions of the atomic scattering centers (pins), while blue dots represent the locations at which the probe particle experienced elastic collisions during simulated trajectories.

These calibration efforts revealed that the chosen set of parameters is both robust and efficient: small enough to ensure fast simulations yet large enough to extract informative and reproducible patterns from a given structure. The replication factor, number of launches, and duration of each trajectory were chosen to optimize the trade-off between simulation cost and fingerprint resolution. All subsequent results and visualizations were obtained under this parametrization, which we found to be sufficient to differentiate between distinct structural organizations using the proposed dynamic descriptors.

Figure 4 illustrates the spatial distribution of collision points for two structural configurations of the graphene sheet generated by different levels of unit cell replication: 2 × 2 × 1 (top) and 5 × 5 × 1 (bottom).

**Figure 4:**
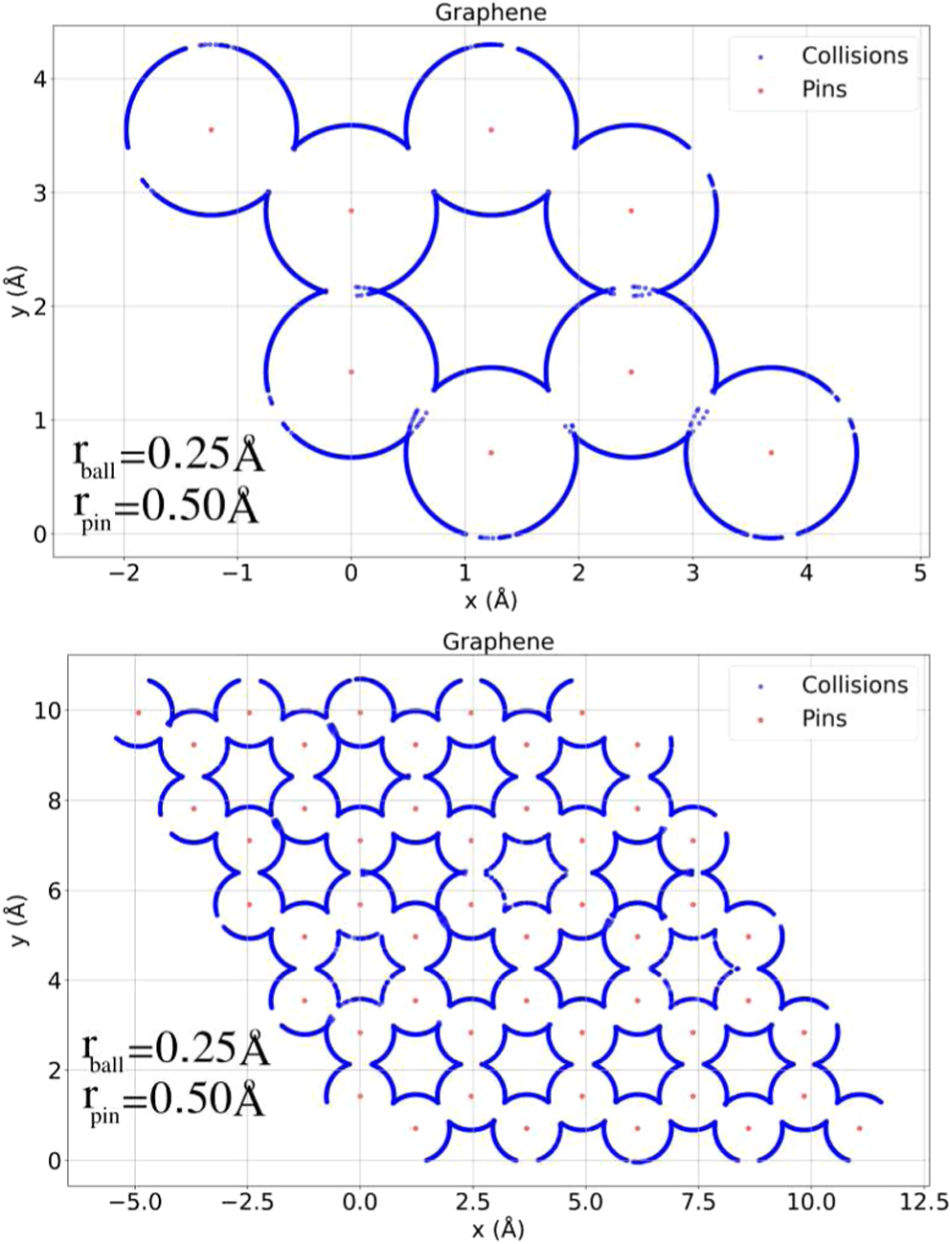
Collision points (blue) overlaid on atomic positions (red) for structures replicated as 2 × 2 × 1 (top) and 5 × 5 × 1 (bottom). The similarity between patterns indicates that the smaller replication already captures the essential structural features for fingerprint analysis.

Despite the substantial difference in the structural size, the two panels reveal remarkably similar collision patterns. The same geometric motifs, such as symmetric scattering zones, directional channels, and characteristic voids, emerge in both configurations. This indicates that the essential features of the structure, from the perspective of dynamic probing via collision statistics, are already well captured in the smaller 2 × 2 × 1 system.

Although the 5 × 5 × 1 replication covers a broader area and contains a larger number of atoms, it does not introduce qualitatively new types of collision events beyond those already observed in the smaller system. Instead, it reproduces and reinforces the same spatial patterns through periodic repetition. This convergence of collision maps suggests that structural fingerprints derived from the 2 × 2 × 1 replication are already representative of the infinite periodic structure. The physical reason lies in the inherently local nature of most collision events, which depend on short- and medium-range atomic arrangements rather than long-range repetition.

This finding is particularly relevant from a practical standpoint. It implies that even small replicated systems are sufficient to perform meaningful structural characterization, provided the unit cell contains representative geometric information. For comparison across different materials or polymorphs, working with compact domains like 2 × 2 × 1 ensures lower computational cost without significant loss in resolution or sensitivity. Consequently, we adopt the smaller replication in benchmark tests and exploratory studies, reserving larger domains only when needed to capture aperiodic features or to reduce edge effects in nonperiodic structures.

Although we verified that a 2 × 2 × 1 replication is sufficient for converged results in the case of graphene, due to its simple periodic lattice, the applicability of the DCF method to more complex structures requires additional attention. Since the descriptor is constructed from statistical distributions of collision events, it is essential that the simulation cell includes all distinct structural motifs, such as different ring sizes, pore geometries, or topological features present in the material. Therefore, before applying DCF to a given system, we recommend verifying that the replication is large enough to reproduce the full set of structural elements characteristic of the material. This ensures that the resulting fingerprint reflects the system’s true geometric and symmetry content rather than an incomplete or biased subset. In materials such as phagraphene or CEY-graphene, where multiple pore types coexist, replication must be chosen accordingly to guarantee descriptor completeness.

Thus, Figure 4 demonstrates not only the consistency and robustness of the dynamic collision fingerprint method but also its efficiency: meaningful results can be obtained from small-scale simulations, reinforcing the method’s suitability for high-throughput applications and comparative structural screening.

Figure 5 presents a comparison of statistical outputs from the collision simulations performed on two structural configurations: a small replicated domain (2 × 2 × 1, top row) and a larger domain (5 × 5 × 1, bottom row). On the left, we show the histograms of free path lengths, that is, the distances traveled by the particle between successive collisions. On the right, we display the corresponding angular order spectra obtained via Fourier transform of the angular distribution of collisions. The histograms reveal a strikingly similar shape in both configurations. A prominent peak is observed at very short distances (near zero), followed by a broad secondary structure peaking around 1.25 to 1.5 Å. The initial sharp peak corresponds to near-immediate recollisions, typically occurring in narrow or crowded regions of the atomic network, where particles quickly bounce back after grazing a nearby pin. This is especially common in dense atomic arrangements, such as those found in hexagonal lattices such as graphene, where atoms are closely spaced and form repeating triangular motifs. These motifs create constrained angular corridors that promote short path recollisions.

**Figure 5:**
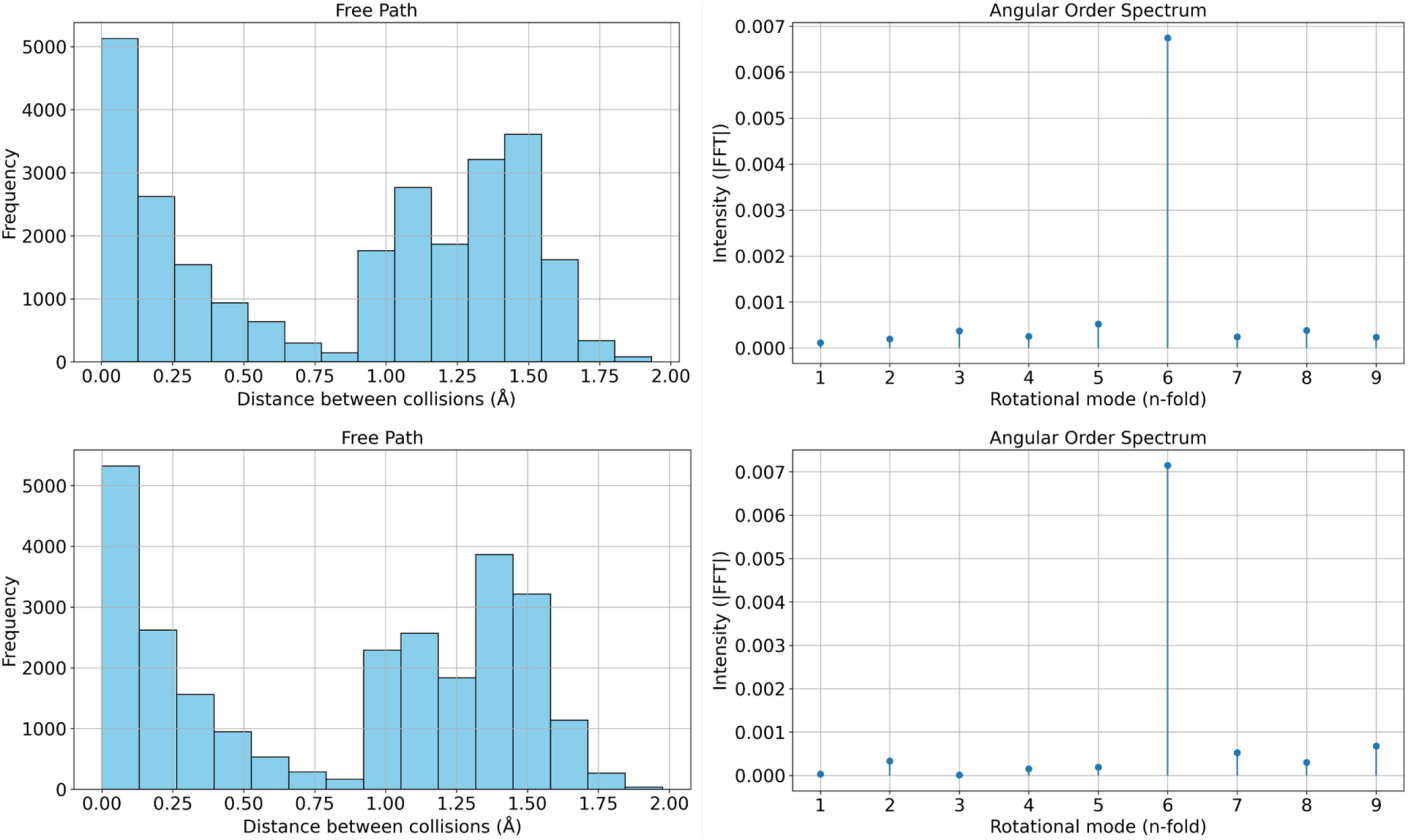
Left: histogram of free path lengths between successive collisions for the 2 × 2 × 1 (top) and 5 × 5 × 1 (bottom) configurations. Right: angular order spectrum obtained from the Fourier transform of the collision angle distribution. The consistency across system sizes demonstrates that essential geometric information is already captured at small scales.

The broader secondary peak arises from larger-scale free paths between atoms located on opposite sides of such motifs. In graphene, the hexagonal symmetry and high connectivity generate multiple preferential scattering directions separated by characteristic distances that fall in this 1.25 Å/1.5 Å range. The resemblance of the histograms across system sizes confirms that the key structural features, such as local connectivity and spatial periodicity, are already captured by the smaller replication. Thus, larger domains merely reinforce the same statistical trends without altering the underlying distribution.

The angular spectra, shown on the right, further support this conclusion. Both configurations exhibit a dominant peak at the 6-fold harmonic, characteristic of hexagonal rotational symmetry, which is the hallmark of the graphene lattice. This clear signal in the Fourier spectrum emerges from the regular recurrence of collision angles aligned with the lattice’s 60^*o*^ periodicity. The remaining harmonics have much lower intensity, reinforcing the notion that the angular scattering behavior is highly structured and dominated by 6-fold order.

Although not shown explicitly, the histogram of relaxation times, i.e., the time intervals between collisions, was also computed and found to mirror the shape of the free path histogram. This correspondence is expected, given that the particle travels at a fixed velocity (determined by the thermal kinetic energy at 300 K). Therefore, the time between collisions τ is directly proportional to the traveled distance λ via τ = λ/*v*, and both distributions retain the same form, scaled by a constant (see relaxation times in the Supporting Information).

Together, these results validate the robustness of the dynamic collision fingerprint method. Both small and larger replications produce equivalent statistical patterns, reinforcing the conclusion that minimal domains, such as 2 × 2 × 1, are sufficient for accurate structural characterization. Moreover, the strong 6-fold signal in the angular spectrum confirms that the method effectively captures intrinsic symmetries in materials like graphene, where directional order is deeply encoded in the atomic topology.

The quantitative results obtained from the simulations further reinforce the consistency and robustness of the dynamic collision fingerprint (DCF) methodology. For the smaller configuration (2 × 2 × 1), the mean free path was found to be λ = 0.848 Å, with a corresponding mean relaxation time τ = 3.731 ps, yielding an effective diffusion coefficient of *D* = 0.048 Å^2^/ps. For the larger configuration (5 × 5 × 1), these values are nearly identical: λ = 0.850 Å, τ = 3.740 ps, and *D* = 0.048 Å^2^/ps. The small absolute differences between the two cases, all within 2–3%, are well within the expected statistical variation given the stochastic nature of the simulations and finite sampling.

These results clearly demonstrate that the essential transport characteristics captured by the model, such as how freely a particle navigates the structure and the average time scale between scattering events, converge rapidly with system size. Importantly, this convergence is achieved already in the 2 × 2 × 1 case, indicating that even minimal replicates suffice to generate reliable descriptors for structural comparison. The implication is powerful: it becomes feasible to perform high-fidelity fingerprinting on minimal structures, significantly reducing the computational cost without compromising accuracy.

In terms of angular information, the entropy values are also consistent between both configurations. The absolute angular entropy is *S* = 0.293, with a normalized value of approximately 0.1015 in both cases. These relatively low entropy values indicate a high degree of directional order in the angular distribution of collisions, which is consistent with the underlying symmetry of the structure. The agreement of entropy across system sizes once again confirms that the angular statistics are not strongly size-dependent, provided that the unit cell itself contains the relevant geometric motifs. The Fourier-based angular order spectrum reveals a dominant 6-fold component in both cases, with intensities of 0.144 and 0.143, for 2 × 2 × 1 and 5 × 5 × 1 replications, respectively.

Taken together, the agreement in diffusion metrics, entropy values, and angular order between the two configurations provides strong evidence for the internal consistency and resolution of the DCF method. The fingerprints extracted are not only quantitatively stable but also qualitatively rich, sensitive enough to detect subtle structural symmetries, yet robust to small-scale variations or domain size. This validates the approach as a practical and reliable tool for characterizing 2D materials, especially in contexts where traditional descriptors fall short or where computational resources are limited.

Continuing the analysis, Figure 6 compares two structural variants of graphene: phagraphene (top row)^51^ and CEY-graphene (bottom row),^52^ both simulated using a 2 × 2 × 1 replication. These systems were selected due to their significant topological differences from pristine graphene, allowing us to evaluate how the collision-based model responds to structurally distinct environments.

**Figure 6:**
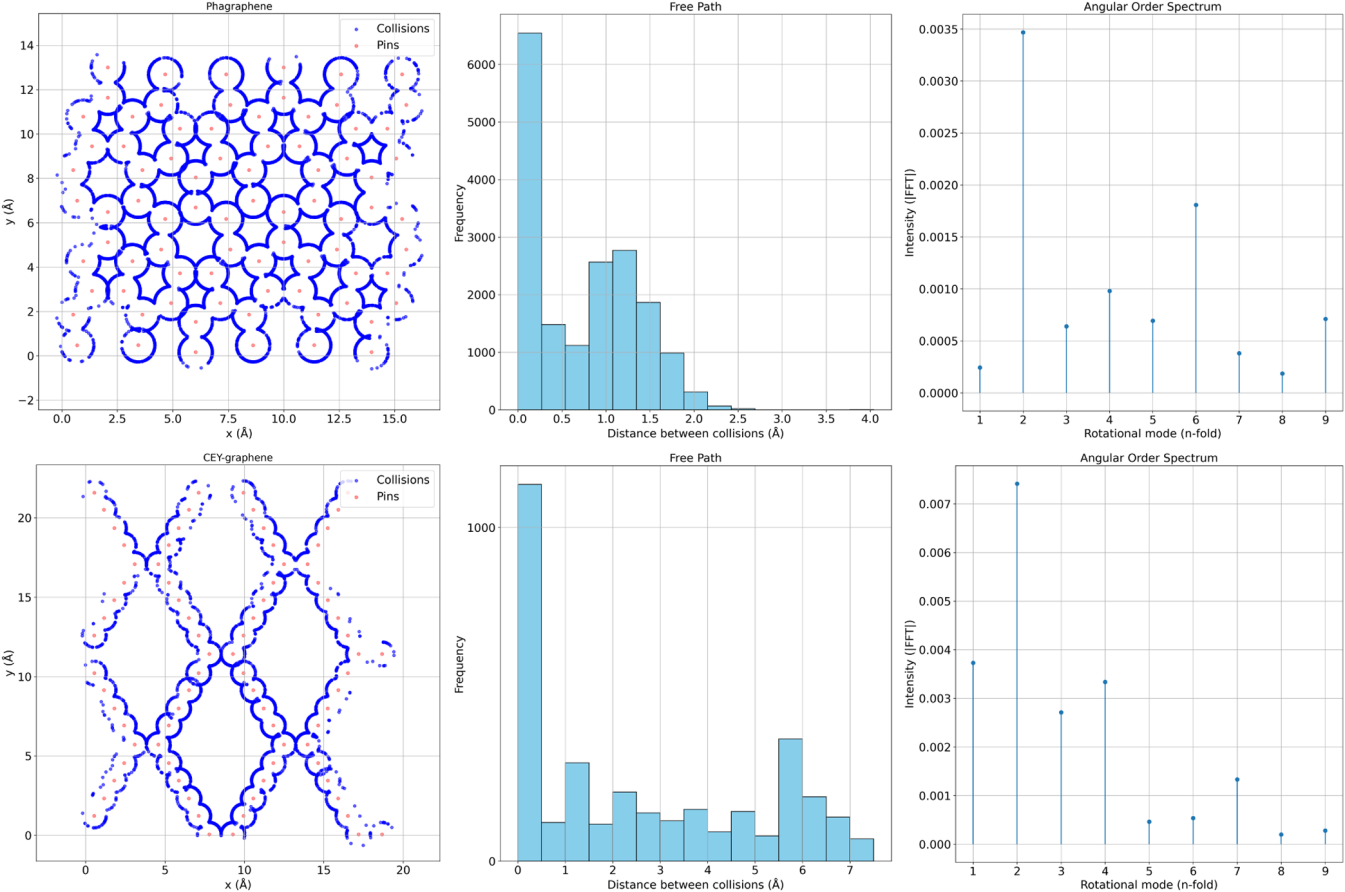
Top: phagraphene; bottom: CEY-graphene. Left: collision maps (blue) over atomic sites (red); center: histograms of free path lengths; right: angular order spectra. While phagraphene exhibits short paths and no dominant angular order, CEY-graphene shows long paths and weak 2-fold symmetry, consistent with its porous and anisotropic atomic structure.

On the left, the spatial maps of collision points reveal clear contrasts. In phagraphene, collisions are dense and relatively uniformly distributed around the atomic sites, illustrating well-defined contours that follow the internal geometry of the material. This structure consists of carbon rings with five, six, and seven members, breaking the ideal hexagonal symmetry and introducing local distortions. Nonetheless, the collision distribution remains compact and isotropic, reflecting a tightly connected atomic network. In contrast, CEY-graphene displays a highly anisotropic pattern with distinct linear channels of collisions and large empty regions in between. This is consistent with its highly porous architecture, which promotes long, unobstructed paths for the particles between collisions.

The central histograms of free path lengths support these visual observations. Phagraphene shows a sharp peak at very short distances (<0.5 Å), followed by a steep decay, which corresponds to its dense atomic packing. CEY-graphene, on the other hand, exhibits a broad, multimodal distribution with free paths extending up to 7 Å. This behavior is a direct consequence of its larger structural voids and open framework. Quantitatively, the mean free path in phagraphene is λ = 0.784 Å, compared to λ = 3.350 Å in CEY-graphene. Accordingly, the effective diffusion coefficient in CEY-graphene is more than three times higher, indicating a much less restrictive environment for particle transport.

Interestingly, despite their differences in dynamic behavior, both systems exhibit similar values of angular entropy (*S* ≈ 0.293, normalized ≈ 0.101), suggesting a lack of pronounced angular order in both cases. This is confirmed by the angular order spectra shown on the right. For phagraphene, the spectrum is relatively flat, with only a weak 2-fold component (intensity ≈ 0.069), insufficient to classify it as angularly ordered. CEY-graphene, however, shows a more pronounced 2-fold peak (intensity ≈ 0.184), qualifying it as an ordered structure with weak 2-fold symmetry. This weak directional order likely arises from the mirror-like alignment of its linear porous channels, which introduces a repeating directional motif despite the overall lack of rotational symmetry.

These results demonstrate that the DCF framework is capable of capturing both global transport characteristics (through metrics such as λ and *D*) and local directional symmetries, even in structurally unconventional systems. In phagraphene, the absence of dominant motifs is reflected in a disordered angular response, while in CEY-graphene, anisotropy and pore alignment manifest as weak, yet detectable, orientational order. This confirms the versatility of the method in distinguishing not only between crystalline and amorphous-like materials but also between distinct classes of ordered yet nonperiodic 2D structures.

In Figure 7, we examine the case of hexagonal boron nitride (h-BN),^53^ a 2D material structurally analogous to graphene but composed of alternating boron and nitrogen atoms, each with distinct atomic radii. This distinction is immediately visible in the left panel, where the circular scattering centers (pins) are drawn with radii proportional to the atomic sizes. Boron and nitrogen, although occupying equivalent lattice positions in terms of symmetry, contribute asymmetrically to the collision dynamics due to their differing pin radii, an effect that subtly distorts the local collision landscape.

**Figure 7:**
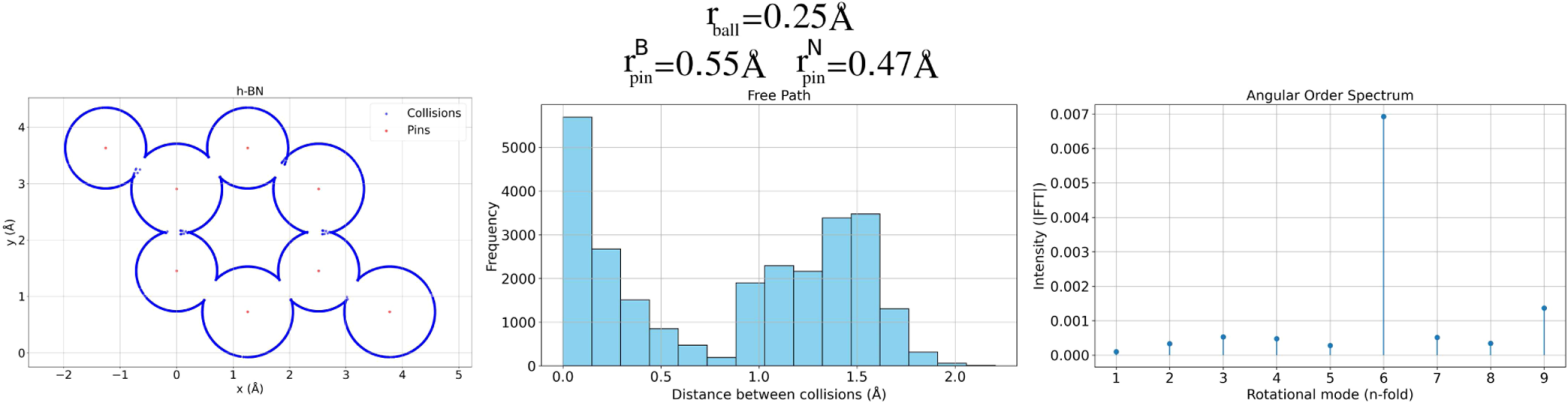
Left: Collision map for h-BN showing radius differences between B and N scatterers. Center: histogram of free paths; right: angular order spectrum. Despite asymmetric pin sizes, the system retains a collision profile similar to that of graphene, with a dominant 6-fold spectral component and comparable diffusion characteristics.

Despite this intrinsic asymmetry, the overall dynamics remains remarkably similar to those observed in pristine graphene. The histogram of free paths (center) presents a bimodal-like profile, with a high-frequency peak at short distances and a broader secondary distribution around 1.5 Å. This behavior closely resembles that of graphene and reflects the hexagonal lattice’s consistent spacing between neighboring atoms, even when the effective collision radius differs slightly. The mean free path is measured at λ = 0.854 Å, and the mean relaxation time is τ = 3.756 ps, yielding an effective diffusion coefficient of *D* = 0.0486 Å^2^/ps. These values are nearly indistinguishable from those found in graphene, indicating that the transport behavior is dominated by the geometric lattice itself rather than small differences in the atomic size.

The angular entropy, *S* = 0.2935 (normalized: 0.1015), is also comparable to that of graphene, indicating a similar level of directional regularity in the collision angles. The angular order spectrum (right) exhibits a clear dominant peak at the 6-fold harmonic, with an intensity of 0.1385. While slightly lower than in graphene, this still supports the classification of h-BN as an ordered structure with weak 6-fold symmetry. The presence of the peak confirms that the hexagonal symmetry of the underlying lattice persists in the dynamic response, even with asymmetrically sized atomic scatterers.

These results suggest that although the use of element-specific pin radii introduces subtle local asymmetries, it does not significantly alter the global statistical behavior of the system, at least for materials with symmetric crystal lattices like h-BN. The similarity in transport descriptors (λ, τ, *D*) and in angular features (entropy and spectral peak) underscores the robustness of the DCF method in capturing meaningful structural information while remaining largely insensitive to small perturbations introduced by chemical composition.

This insensitivity is advantageous in comparative studies, as it allows structurally similar materials, like graphene and h-BN, to be distinguished based on geometry-driven fingerprints, rather than requiring explicit chemical detail. Nonetheless, the model still captures asymmetry when it becomes structurally significant, as seen in earlier examples like CEY-graphene. Here, however, the lattice symmetry dominates over the radius asymmetry, reinforcing the conclusion that geometry and topology are the primary determinants of the dynamic response in regular 2D materials.

Figure 8 marks a conceptual and methodological advancement in the development of the dynamic collision fingerprint (DCF) by showing how the statistical outputs of the model can be quantitatively encoded into a structured, machine-readable descriptor. Until this point, we have demonstrated that the trajectory-based model captures the meaningful physical and geometric properties of 2D materials. Here, we move one step further by fitting the distribution of free paths using a bimodal Gaussian mixture model (GMM) and incorporating this alongside dynamic and angular features into a comprehensive structural fingerprint.

**Figure 8:**
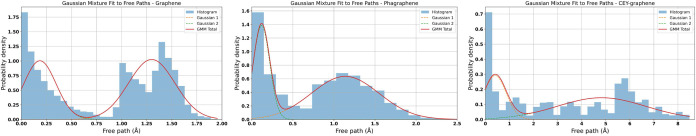
Gaussian Mixture Model (GMM) fit to the free path histograms for graphene (left), phagraphene (center), and CEY-graphene (right). The extracted GMM parameters, along with transport statistics, angular entropy, and Fourier mode intensities, compose the proposed dynamic collision fingerprint descriptor. This structured output can be used directly for structural classification or integrated into broader materials discovery platforms.

In Figure 8, we show the GMM fits for three representative systems: graphene (left), phagraphene (center), and CEY-graphene (right). The histograms represent the empirical distribution of free path lengths extracted from the simulated trajectories, while the red curve corresponds to the sum of two fitted Gaussians. These two modes can be interpreted physically: the first component generally captures short-range scattering events, associated with densely packed atomic arrangements or immediate recollisions, whereas the second component reflects longer-range flights governed by structural pores, channels, or intermediate voids.

In graphene, the bimodal shape is evident and well captured by the model, with two distinct peaks, one near the origin and another around 1.3–1.4 Å. Phagraphene exhibits a similarly decaying distribution but with broader overlap between components, reflecting its geometrically disordered (though still dense) network of 5-, 6-, and 7-membered rings. In contrast, CEY-graphene shows a completely different profile: the histogram is wide and flat, spanning up to 7 Å, with the GMM identifying long, porous corridors as dominant contributors to the second Gaussian mode. The success of this fitting approach supports its utility as a compact representation of the structural scattering landscape.

To formalize this representation, we extract a vector of numerical features from each simulation, including:

- Transport descriptors: mean free path, relaxation time, and effective diffusivity;
- Statistical moments: standard deviation, median, skewness, and kurtosis of the path distribution;
- GMM parameters: means and weights of the two Gaussian components;
- Angular descriptors: absolute and normalized angular entropy;
- Fourier intensities: the magnitude of each harmonic mode from 1-fold to 9-fold in the angular order spectrum.

This descriptor, entirely derived from a simple mechanical model, is highly compact, low-dimensional, and rich in information. It is capable of distinguishing between materials with different atomic arrangements, symmetries, and transport behavior. Additionally, the descriptor is designed to be interpretable, as each feature corresponds to a measurable physical or geometric quantity, and modular, allowing integration with other feature sets, such as those from libraries like ASE, or to be used as input in machine learning pipelines for classification, clustering, or regression.

As discussed earlier, we propose that each particle in the model should follow a trajectory with *N*_step_ = 10,000 and that 100 particles (*N*_launches_ = 100) with distinct initial conditions should be launched from a given point of the structure. For the case of phagraphene, using the suggested parameters, the total simulation time with our code is approximately 4 min. While acceptable for only calculation, this duration can become a limiting factor when applying the method to large data sets involving hundreds or even thousands of structures. To address this, we recommend reducing the number of steps *N*_step_ where appropriate. For instance, in the case of phagraphene, using *N*_step_ = 1000 slightly alters the descriptor values but has a minimal impact on the overall fitting or trend analysis. Moreover, this change reduces the computation time to just 25 s, significantly improving efficiency with only a modest trade-off in accuracy. Based on these considerations, we suggest testing different values of *N*_step_ and verifying, through benchmark simulations, whether the accuracy of the machine learning model is preserved or only slightly reduced. In general, the user has full flexibility to modify any parameter in the code. However, for carbon-based systems and similar structures, we recommend adjusting primarily the number of steps (*N*_step_), the number of launches (*N*_launches_), and the supercell replication factor, which may be required to adequately represent all rings and pores present in the system. We recommend keeping all other parameters unchanged.

Furthermore, this output descriptor can serve not only as a standalone structural signature but also as a complementary layer in hybrid frameworks that combine geometric, electronic, and topological information. We provide the full Python implementation used to compute this fingerprint, enabling researchers to apply the DCF framework to any 2D structure in the CIF format and to integrate it into their materials informatics workflows.

In the code provided to compute the structural parameters using the DCF method, we also include functionalities to replicate the input structure and introduce a random distribution of vacancies, enabling the investigation of defect-induced effects on the structural descriptors.

In summary, the proposed dynamic collision fingerprint (DCF) descriptors represent a novel and physically grounded approach to structural characterization, offering a compact, interpretable, and versatile tool with strong potential for applications in 2D materials science—particularly in the analysis, comparison, and discovery of carbon-based systems and other atomically thin architectures. Notably, all calculations were performed on a standard personal computer, without the need for high-performance computing resources, highlighting the method’s simplicity, efficiency, and accessibility.

## Conclusions

We have introduced a novel approach to structural characterization based on the dynamics of classical particle scattering in 2D atomic lattices. Inspired by the Drude–Galton model, dynamic collision fingerprint (DCF) captures how a structure responds to mechanical perturbations, generating a set of descriptors derived from free path statistics, angular distributions, and their spectral features. Through extensive simulations on diverse 2D systems—including graphene, phagraphene, CEY-graphene, and h-BN—we demonstrated that DCFs are sensitive to lattice symmetry, porosity, and disorder while remaining computationally efficient and easily generalizable.

Importantly, we showed that even small replicated domains are sufficient to extract meaningful fingerprints and that these descriptors can be compactly encoded via statistical analysis and Gaussian mixture modeling. The resulting feature vectors are interpretable, low-cost, and well-suited for integration into machine learning workflows or as complements to conventional structural descriptors.

The DCF framework opens up a new perspective on structure–property relationships in materials science, particularly for 2D systems where geometry plays a dominant role. Its ability to resolve subtle differences in order, symmetry, and connectivity using purely geometric and mechanical principles makes it a promising tool for the high-throughput screening, classification, and discovery of novel materials.

Beyond its immediate application for structural characterization, DCF opens promising perspectives for materials discovery and property prediction in two-dimensional systems. Given that DCF encodes geometric and symmetry-related features through dynamic interaction patterns, it may serve as a robust input for machine learning models aiming to predict mechanical, electronic, or transport properties. In particular, its sensitivity to both local and long-range order, combined with its robustness to disorder and minimal parametrization, suggests that the DCF can complement or even outperform traditional descriptors, especially in complex, defective, or nonperiodic 2D materials. Notably, our framework readily allows the introduction of controlled disorder, such as random vacancies, providing a simple yet powerful tool to investigate how structural imperfections influence orientational order and other key properties. A systematic comparison between DCF and conventional descriptors in property prediction tasks constitutes an exciting avenue for future research, which we intend to pursue.

## Supplementary Material



## Data Availability

All simulation scripts developed in this work are publicly available at: https://github.com/tromer-unb/DCF. The repository includes two main codes: run.py, which simulates particle trajectories for a single system in periodic atomic structures to compute transport properties (mean free path, relaxation time, diffusivity, and angular entropy); and run_descriptor.py, which automates the extraction of structural descriptors from multiple systems. Both codes require.cif structure files as input and include example parameter files and preprocessing tools.

## Notes

Histogram of relaxation times for all structures considered in the manuscript (graphene, phagraphene, cey-graphene, and BN) (PDF)

## References

[1] Goodall R. E. A., Lee A. A. (2019). Predicting materials
properties without crystal structure:
Deep representation learning from stoichiometry. Nat Commun..

[2] Aliyev E., Filiz V., Khan M. M., Lee Y. J., Abetz C., Abetz V. (2019). Structural Characterization
of Graphene Oxide: Surface Functional
Groups and Fractionated Oxidative Debris. Nanomaterials.

[3] Wieder B. J., Bradlyn B., Cano J., Wang Z., Vergniory M. G., Elcoro L., Soluyanov A. A., Felser C., Neupert T., Regnault N., Bernevig B. A. (2022). Topological
materials discovery from
crystal symmetry. Nat. Rev. Mater..

[4] Ling X., Wang H., Huang S., Xia F., Dresselhaus M. S. (2015). The renaissance
of black phosphorus. Proc. Natl. Acad. Sci.
U.S.A..

[5] Li L. H., Santos E. J., Xing T., Cappelluti E., Roldán R., Chen Y., Okada S. (2015). Dielectric screening
in atomically thin boron nitride nanosheets. Nano Lett..

[6] Liu H., Neal A., Zhu Z., Luo Z., Xu X., Tománek D., Ye P. (2014). Phosphorene: An Unexplored
2D Semiconductor
with a High Hole Mobility. ACS Nano.

[7] Lee C., Wei X., Kysar J. W., Hone J. (2008). Measurement of the Elastic Properties
and Intrinsic Strength of Monolayer Graphene. Science.

[8] Lopez-Sanchez O., Lembke D., Kayci M., Radenovic A., Kis A. (2013). Ultrasensitive photodetectors based on monolayer MoS2. Nat. Nanotechnol..

[9] Dean C. R., Young A., Meric I., Lee C., Wang L., Sorgenfrei S., Watanabe K., Taniguchi T., Kim P., Shepard K., Hone J. (2010). Boron nitride substrates for high-quality
graphene electronics. Nat. Nanotechnol..

[10] Ji W., Jin C., Xu H., Gao J. R., Gröblacher S., Urbach H. P., Adam A. J. L. (2023). Recent
advances in metasurface design
and quantum optics applications with machine learning, physics-informed
neural networks, and topology optimization methods. Light:Sci. Appl..

[11] Yin K., Hsiang E., Hsiang E.-L., Zou J., Li Y., Yang Z., Yang Q., Lai P.-C., Lin C.-L., Wu S. (2022). Advanced liquid crystal
devices for augmented reality
and virtual reality displays: principles and applications. Light:Sci. Appl..

[12] Novoselov K. S., Geim A., Morozov S., Jiang D., Zhang Y., Dubonos S., Grigorieva I., Firsov A. (2004). Electric Field Effect
in Atomically Thin Carbon Films. Science.

[13] Geim A. K., Novoselov K. (2007). The rise of
graphene. Nat. Mater..

[14] Kývala L., Hijes P. M. D., Dellago C. (2024). Unsupervised
identification of crystal
defects from atomistic potential descriptors. npj Comput. Mater..

[15] Zhang, R. ; Rong, F. ; Lai, G. ; Wu, G. ; Ye, Y. ; Zheng, J. Machine learning descriptors for crystal materials: applications in Ni-rich layered cathode and lithium anode materials for high-energy-density lithium batteries J. Mater. Inf. 2024, 4, 10.20517/jmi.2024.22.

[16] Galanakis N., Tuckerman M. (2024). Rapid prediction of molecular crystal
structures using
simple topological and physical descriptors. Nat. Commun..

[17] Fayos J., Fayos J. (2009). Molecular Crystal Prediction
Approach by Molecular Similarity: Data
Mining on Molecular Aggregation Predictors and Crystal Descriptors. Cryst. Growth Des..

[18] Inada Y., Katsura Y., Kumagai M., Kimura K. (2021). Atomic descriptors
generated from coordination polyhedra in crystal structures. Sci. Technol. Adv. Mater.:Methods.

[19] Jäger M. O. J., Jäger M. O. J., Morooka E. V., Canova F. F., Rinke P., Himanen L., Foster A. S. (2018). Machine learning
hydrogen adsorption on nanoclusters through structural descriptors. npj Comput. Mater..

[20] Minjeong C., Cha M., Emre E. S. T., Turali-Emre E. S., Xiao X., Kim J.-Y., Bogdan P., VanEpps J. S., Violi A., Kotov N. A. (2022). Unifying
structural descriptors for biological and bioinspired nanoscale complexes. Nat. Comput. Sci..

[21] Uzal-Varela R., Pérez-Fernández F., Valencia L., Rodríguez-Rodríguez A., Platas-Iglesias C., Platas-Iglesias C., Caravan P., Esteban-Gómez D., Esteban-Gómez D. (2022). Thermodynamic Stability of Mn­(II) Complexes with Aminocarboxylate
Ligands Analyzed Using Structural Descriptors. Inorg. Chem..

[22] Gong K., Olivetti E. (2021). Development of structural
descriptors to predict dissolution
rate of volcanic glasses: molecular dynamic simulations. J. Am. Ceram. Soc..

[23] Sciortino G., Garribba E., Savic I., Pedregal J. R.-G., Maréchal J., Maréchal J.-D. (2019). Simple Coordination Geometry Descriptors Allow to Accurately
Predict Metal-Binding Sites in Proteins. ACS
Omega.

[24] Kute M., Deng Z., Chae S., Kioupakis E. (2021). Cation-size
mismatch as a predictive descriptor for structural distortion, configurational
disorder, and valence-band splitting in II-IV-N2 semiconductors. Appl. Phys. Lett..

[25] Liu J., Wang P., Luan J.-H., Chen J., Cai P., Chen J., Lu X., Fan Y., Yu Z., Chou K. (2024). VASE: A High-Entropy Alloy Short-Range
Order Structural Descriptor
for Machine Learning. J. Chem. Theory Comput..

[26] Han X.-B., Han X., Jing C.-Q., Jing C., Zu H.-Y., Zhang W. (2022). Structural
Descriptors to Correlate Pb Ion Displacement and Broadband Emission
in 2D Halide Perovskites. J. Am. Chem. Soc..

[27] Ng W. L., Goh G. L., Goh G. D., Sheuan J. T. J., Yeong W. Y. (2024). Progress
and Opportunities for Machine Learning in Materials and Processes
of Additive Manufacturing. Adv. Mater..

[28] Murdock R. J., Murdock R. J., Kauwe S. K., Wang A. Y.-T., Wang A., Sparks T. D. (2020). Is Domain Knowledge Necessary for
Machine Learning
Materials Properties. Integr. Mater. Manuf.
Innovation.

[29] Mortazavi B. (2024). Recent Advances
in Machine Learning-Assisted Multiscale Design of Energy Materials. Adv. Energy Mater..

[30] Chandonia J.-M., Guan L., Lin S., Yu C., Fox N., Fox N. K., Brenner S. E. (2021). SCOPe: improvements
to the structural
classification of proteins - extended database to facilitate variant
interpretation and machine learning. Nucleic
Acids Res..

[31] Miyajima Y., Mochizuki M. (2023). Machine-learning detection of the
Berezinskii-Kosterlitz-Thouless
transition and the second-order phase transition in XXZ models. Phys. Rev. B.

[32] Cheng B. (2024). Cartesian
atomic cluster expansion for machine learning interatomic potentials. npj Comput. Mater..

[33] Watanabe N., Hori Y., Sugisawa H., Ida T., Shoji M., Shigeta Y. (2024). A machine learning potential construction
based on
radial distribution function sampling. J. Comput.
Chem..

[34] Liebetrau M., Dorenkamp Y., Bünermann O., Behler J. (2023). Hydrogen atom scattering
at the Al2O3(0001) surface: a combined experimental and theoretical
study. Phys. Chem., Chem. Phys..

[35] Simon A., Belloni L., Borgis D., Oettel M. (2025). The orientational structure
of a model patchy particle fluid: Simulations, integral equations,
density functional theory, and machine learning. J. Chem. Phys..

[36] Langer M. F., Pozdnyakov S., Ceriotti M. (2024). Probing the effects of broken symmetries
in machine learning. Mach. Learn. Sci. Technol..

[37] Liu X., Lu W., Tu W., Shen J. (2022). Identifying stress-induced
heterogeneity
in Cu20Zr20Ni20Ti20Pd20 high-entropy metallic glass from machine learning
atomic dynamics. J. Mater. Inf..

[38] Zhang B., Zhang X., Du W., Song Z., Zhang G., Zhang G., Wang Y., Wang Y., Chen X., Jiang J. (2022). Chemistry-informed molecular graph as reaction descriptor
for machine-learned retrosynthesis planning. Proc. Natl. Acad. Sci. U.S.A..

[39] Barnard T., Tseng S., Darby J. P., Bartók A. P., Broo A., Sosso G. C. (2023). Leveraging genetic algorithms to
maximise the predictive capabilities of the SOAP descriptor. Mol. Syst. Des. Eng..

[40] Parsaeifard B., Goedecker S. (2021). Manifolds
of quasi-constant SOAP and ACSF fingerprints
and the resulting failure to machine learn four body interactions. J. Chem. Phys..

[41] Wu W., Qian J., Liang C., Yang J., Ge G.-B., Zhou Q., Guan X.-Q. (2023). GeoDILI: A Robust and Interpretable
Model for Drug-Induced Liver Injury Prediction Using Graph Neural
Network-Based Molecular Geometric Representation. Chem. Res. Toxicol..

[42] Rosenblatt M., Tejavibulya L., Jiang R., Noble S., Scheinost D. (2024). Data leakage
inflates prediction performance in connectome-based machine learning
models. Nat. Commun..

[43] Ernst O. K., Bartol T. M., Sejnowski T. J., Mjolsness E. (2019). Learning moment
closure in reaction-diffusion systems with spatial dynamic Boltzmann
distributions. Phys. Rev. E.

[44] Wang H., Du K., Jiang C., Yang Z., Ren L., Zhang W., Chua S. J., Mei T. (2019). Extended Drude Model
for Intraband-Transition-Induced
Optical Nonlinearity. Phys. Rev. Appl..

[45] Levi, A. F. J. ; Levi, A. F. J. Drude Model 2016.

[46] Gong, J. Galton Board Experiment: Proof of Central Limit Theorem Sci. Technol. Eng., Chem., Environ. Prot. 2024, 1 3 10.61173/z5yb2616.

[47] Qin H., Cheng R., Zhou Y., Tang H. X. (2024). Integrated photonic
Galton board and its application for photon counting. Opt. Quantum.

[48] Zhao J., Zhao J. T., Qi C., Li G., Schmidt M. A., Schmidt M. (2020). An improved spectrophotometric method tests the Einstein–Smoluchowski
equation: a revisit and update. Phys. Chem.
Chem. Phys..

[49] Bisikalo O., Kharchenko V., Kovtun V., Krak I., Pavlov S. V. (2023). Parameterization
of the Stochastic Model for Evaluating Variable Small Data in the
Shannon Entropy Basis. Entropy.

[50] Cooley J. W., Tukey J. W. (1965). An algorithm for the machine calculation of complex
Fourier series. Math. Comput..

[51] López-Bezanilla A., Lopez-Bezanilla A. (2016). Strain-Mediated
Modification of Phagraphene Dirac Cones. J.
Phys. Chem. C.

[52] Makaremi M., Mortazavi B., Singh C. V. (2018). Carbon ene-yne graphyne monolayer
as an outstanding anode material for Li/Na ion batteries. Appl. Mater. Today.

[53] Kim S. M., Hsu A., Park M. H., Chae S. H., Yun S. J., Lee J. S., Cho D.-H., Fang W., Lee C., Palacios T. (2015). Synthesis of large-area multilayer hexagonal
boron nitride for high
material performance. Nat. Commun..

