# Supplementary material for: Dynamic Collision Fingerprints (DCF): Introducing a New Descriptor Linking Lattice Interactions to 2D Structural Data Signatures
Source: J Chem Theory Comput. 2025 Aug 12;21(16):8106–18. doi: 10.1021/acs.jctc.5c00856 (PMC12392443; doi:10.1021/acs.jctc.5c00856)
Supplement: Supplementary file 1 [file ct5c00856_si_001.pdf]

Supporting Information

Dynamic Collision Fingerprints (DCF): Introducing a New Descriptor Linking Lattice Interactions to 2D Structural Data Signatures

Raphael M. Tromer*

University of Brasília, Institute of Physics, Brasília, Federal District, 70910-900, Brazil.

E-mail: raphael.tromer@unb.br

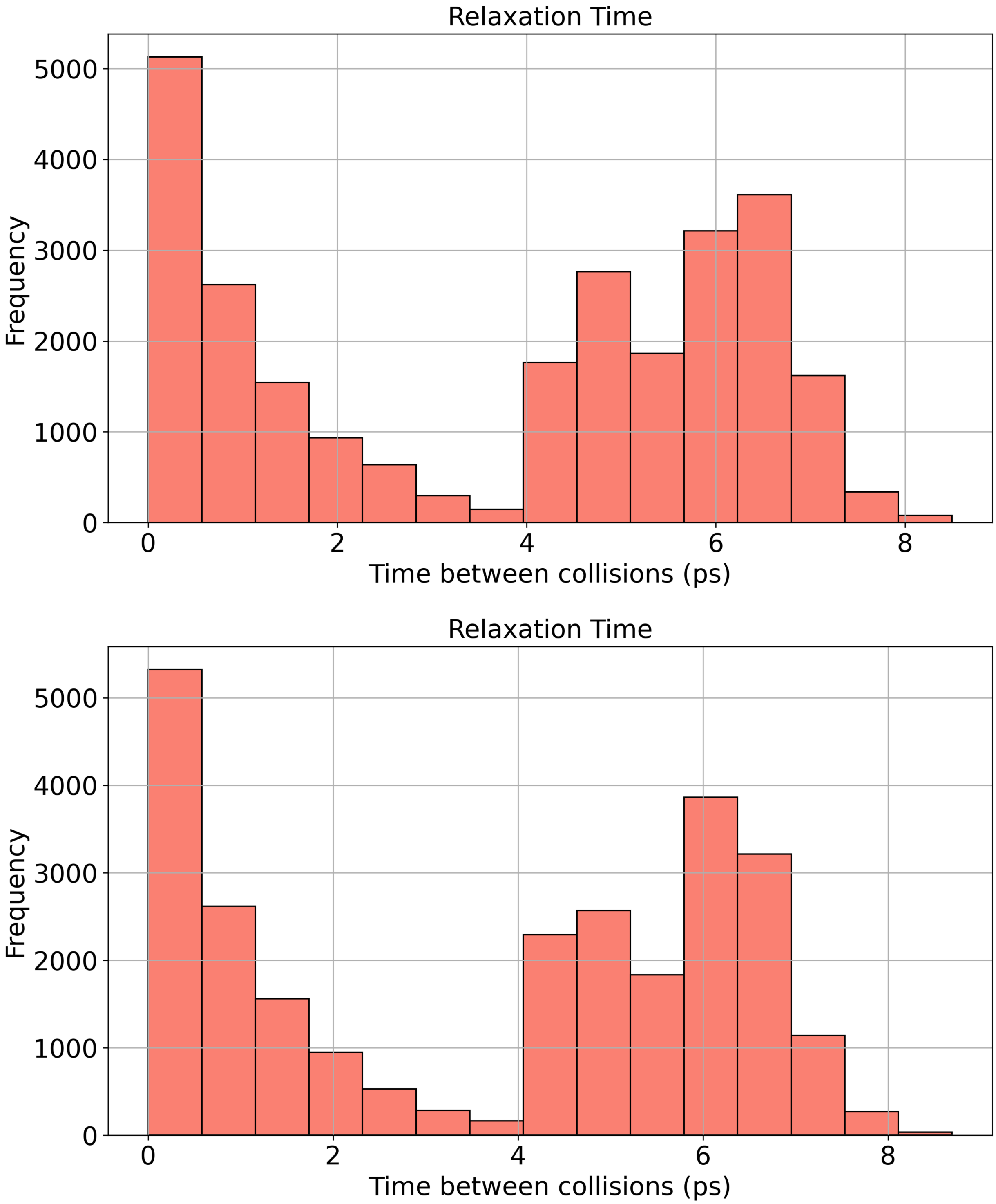

Figure S1: Comparison of relaxation times for graphene modeled with $2 \times 2 \times 1$ and $5 \times 5 \times 1$ supercell replications, shown at the top and bottom, respectively.

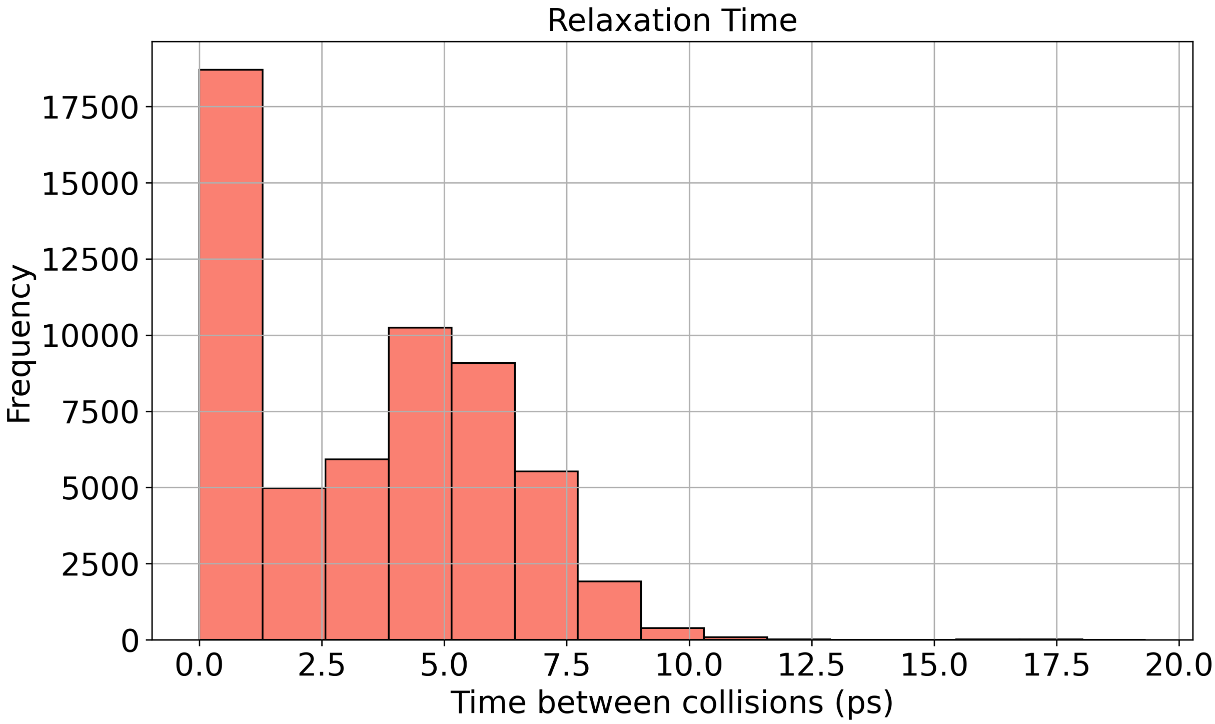

Figure S2: Relaxation time of phagraphene with $2 \times 2 \times 1$ replication.

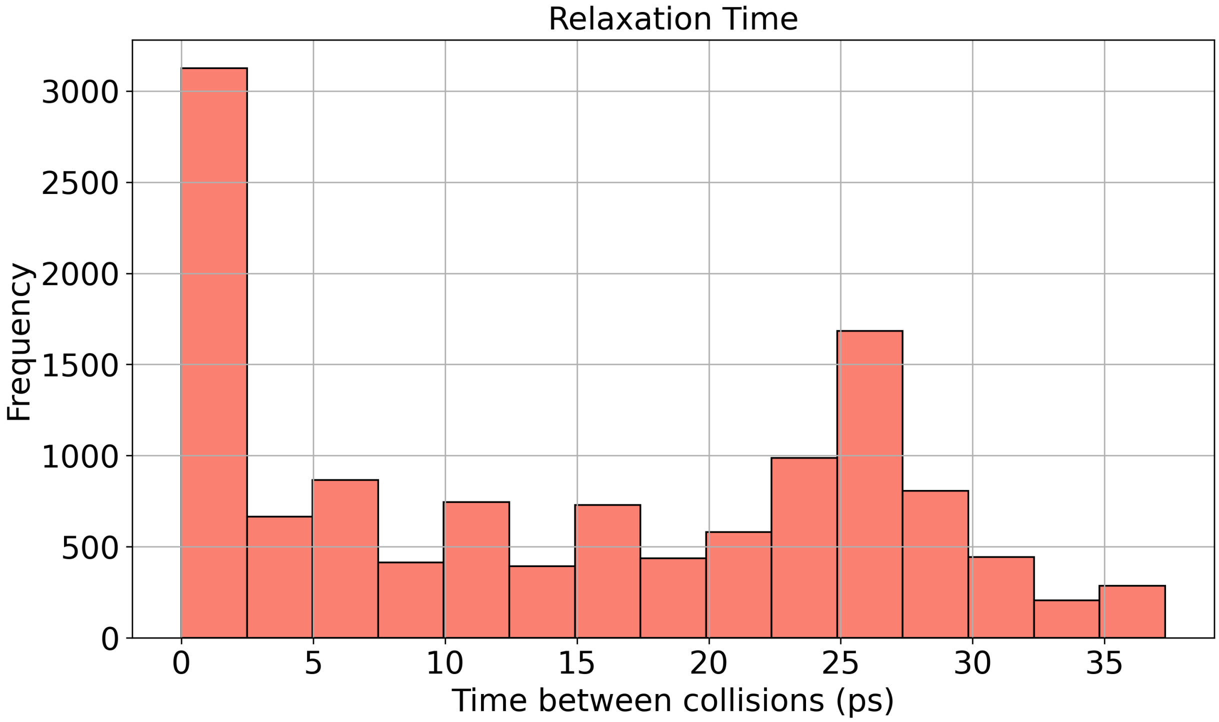

Figure S3: Relaxation time of Cey-phagraphene with $2 \times 2 \times 1$ replication.

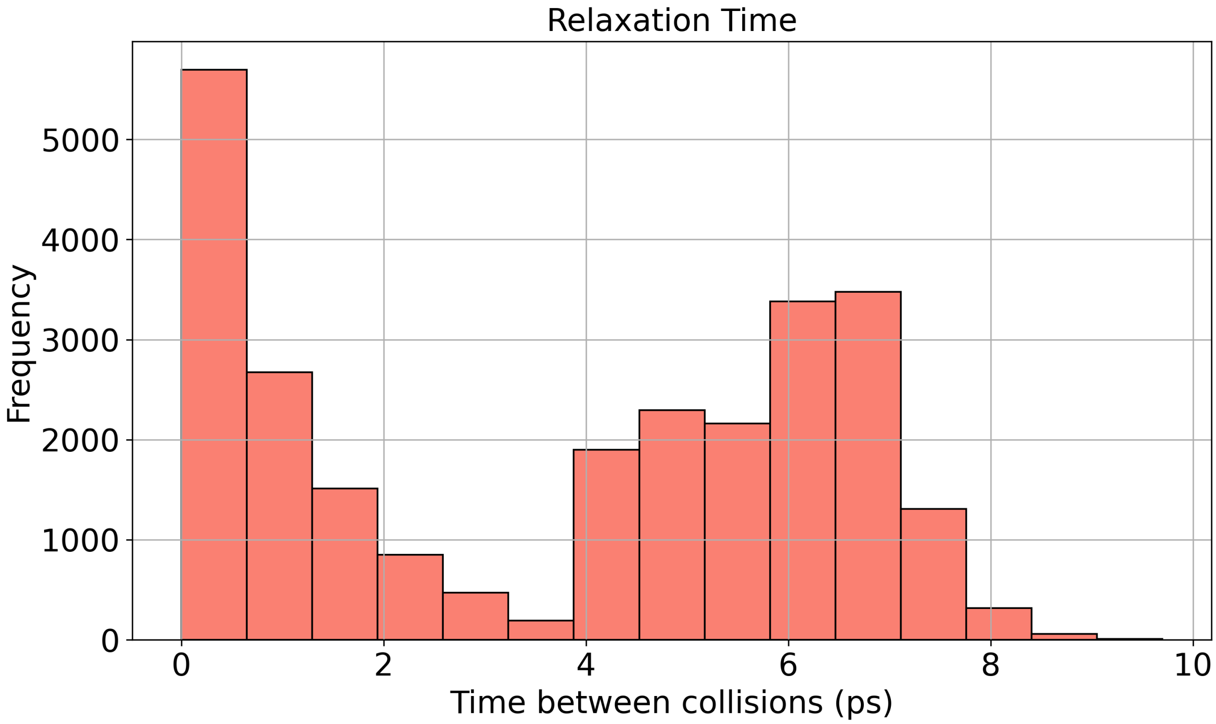

Figure S4: Relaxation time of h-BN with $2 \times 2 \times 1$ replication.
